# Bridging Species with AI: A Cross-Species Deep Learning Model for Fracture Detection and Beyond

**DOI:** 10.3390/bioengineering13020213

**Published:** 2026-02-13

**Authors:** Hanya T. Ahmed, Dagmar Berner, Qianni Zhang, Kristien Verheyen, Francisco Llabres-Diaz, Vanessa G. Peter, Yu-Mei Chang

**Affiliations:** 1 Department of Comparative Biomedical Sciences, Royal Veterinary College, London NW1 0TU, UK; ychang@rvc.ac.uk; 2 Department of Clinical Science and Services, Royal Veterinary College, London NW1 0TU, UK; 3 School of Electronic Engineering and Computer Science, Queen Mary University of London, London E1 4NS, UK; qianni.zhang@qmul.ac.uk; 4 Department of Pathobiology and Population Sciences, Royal Veterinary College, London NW1 0TU, UK; 5 Veterinary Diagnostic Imaging, Rossdales Equine Hospital & Diagnostic Centre, Newmarket CB8 7NN, UK

**Keywords:** detection, medical imaging, transformers, fractures, horse

## Abstract

Fractures are a leading cause of morbidity and mortality in Thoroughbred racehorses, posing a significant threat to their welfare and careers. This study introduces a deep learning model specifically designed to facilitate fracture detection in equine athletes. By leveraging extensive training on human fracture data and refining the model with equine imaging, it highlights the transformative potential of transfer learning across species and medical contexts. This approach is not limited to equine fractures but could be adapted for use in detecting injuries or conditions in other veterinary species and even human healthcare applications. A comprehensive databank of radiographs, sourced from public archives and equine hospitals, was curated to encompass diverse conditions (fracture and non-fracture), ensuring robust pattern recognition. The architecture integrates a Vision Transformer for global context modelling with a ResNet backbone and loss function to optimize local feature extraction and cross-species adaptability. The pipeline achieved 96.7% accuracy for modality classification, 97.2% accuracy for projection recognition, and fracture localization intersection over union values of 0.71–0.84 across equine datasets. This work bridges advancements in human and veterinary medicine, opening pathways for AI-driven solutions that extend beyond fractures, fostering improved diagnostic precision and broader applications across species (felines, canines, etc.). By integrating advanced imaging techniques with AI, this study aims to set a foundation for more comprehensive and versatile health monitoring systems.

## 1. Introduction

In horses, the metacarpophalangeal and metatarsophalangeal joints are referred to as the ‘fetlock’. Fetlock fractures are a leading cause of catastrophic musculoskeletal injury in Thoroughbred racehorses, posing serious risks to animal welfare and racing careers and increasing veterinary costs. These injuries can vary in severity; while simple condylar fractures are often surgically repairable with good outcomes, more complex or comminuted fractures frequently necessitate euthanasia due to the biomechanical complexity of the fetlock joint and the challenges associated with stabilizing the joint under high loading conditions [1,2]. Current diagnostic methods, which are primarily radiographic assessment, are frequently limited by image quality, projection variability, and the difficulty of detecting subtle lesions, especially in high-performance animals under stress [3]. Although advanced imaging such as computed tomography (CT) and magnetic resonance imaging (MRI) is increasingly used in specialist settings, these modalities remain less accessible.

Over the last decade, advances in artificial intelligence (AI) and deep learning (DL) have transformed diagnostic imaging in human medicine. Numerous studies have shown that convolutional neural networks (CNNs) and transformer-based models can perform at the human level in fracture detection, imaging modality classification, and anatomical localization [4,5]. Models trained on large-scale radiographic datasets, such as MURA and CheXpert, have demonstrated strong pattern recognition for a wide range of musculoskeletal and thoracic abnormalities [6,7].

Despite these advances, deep learning applications in the veterinary field are still in their early stages. In contrast to human medicine, where large scale datasets are publicly available and radiographic datasets [8] have catalyzed AI development and benchmarking, there are currently no equivalent open datasets or research competitions (e.g., FastMRI and AIROGS Challenge) in veterinary imaging. Equine radiographs (XR), and veterinary imaging data more generally, remain largely absent from public repositories due to privacy concerns, a lack of standardized imaging protocols, and institutional barriers to data sharing. This scarcity of curated datasets has significantly limited progress in applying deep learning to veterinary diagnostics, despite its demonstrated success in human healthcare. Prior research in veterinary AI work has focused on areas such as lameness detection with wearable/motion sensors, the automated analysis of respiratory sounds, and disease prediction from routine laboratory data [9,10,11]. Furthermore, the distinct anatomical differences between species make it difficult to transfer human-trained models directly into veterinary settings without significant domain adaptation [12].

Transfer learning, which involves repurposing and fine-tuning a model trained on one task or domain for use on another, appears to be a promising approach to closing this data gap. For example, models pretrained on large human imaging cohorts have been successfully adapted to non-human primates with limited species-specific data, improving performance and generalization [13]. However, few studies have investigated this in clinical settings like equine fracture detection.

This study presents a proof-of-concept cross-species deep learning framework for detecting fractures in equine athletes, with initial training based on human radiographic data and performance refined using a curated dataset of equine fetlock radiographs. The architecture combines Vision Transformers (ViTs) and ResNet backbones, as well as a custom loss function, to improve feature learning across anatomical domains. The dataset covers a wide range of clinical conditions, including fracture, post-treatment, and non-fracture, allowing for robust generalization across diverse equine cases.

Beyond equine applications, the model was further validated on feline datasets, highlighting its potential as a foundation for multi-species veterinary diagnostics. The results show strong localization capabilities and diagnostic accuracy, indicating that cross-species transfer learning is viable. This work lays the groundwork for future AI-assisted diagnostics in veterinary medicine, as well as translational models that could inform human clinical tools.

## 2. Materials and Methods

**Figure 1:**
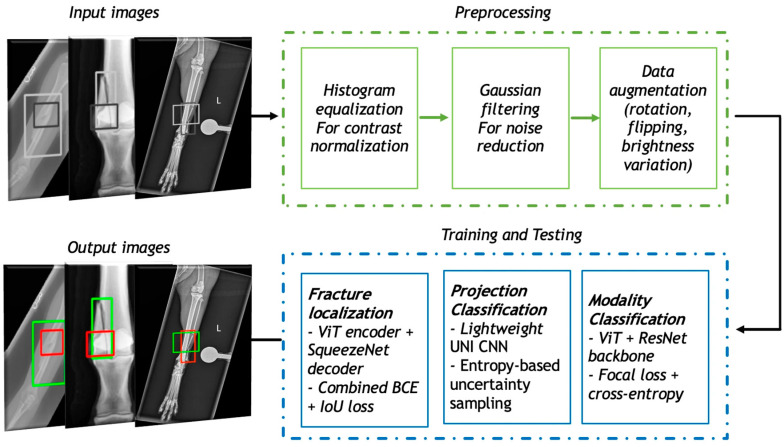
An overview of the cross-species deep learning pipeline for fracture detection. The modular framework includes three stages: modality classification using a ViT-ResNet hybrid, projection classification using a lightweight UNI model with active learning, and fracture localization on DP views for an equine dataset using a ViT–SqueezeNet model trained with a combined BCE + IoU loss. Transfer learning from human radiographs enabled adaptation to equine and feline datasets.

To develop a robust and generalizable model for cross-species fracture detection, we built a deep learning pipeline that included curated datasets, tailored preprocessing steps, and a hybrid model architecture (Figure 1). This section describes the steps taken for data collection, annotation, model development, transfer learning, training and evaluation. Our methodology emphasizes adaptability across imaging modalities and species, drawing on both human and veterinary medical data.

### 2.1. Data Collection

This study used a diverse, cross-species dataset (images) that included equine, feline and human images (Table 1). One hundred equine fetlock imaging cases (67 fracture cases, 33 non-fracture cases) were collated from the published veterinary literature and anonymized radiographs from equine hospital archives. These cases included radiographs (XR), computed tomography (CT) and magnetic resonance imaging (MRI). These images depicted a wide range of conditions, including fracture (acute injury), post-treatment (for example, with implants), and non-fracture (healthy or non-fracture pathology) states. To test the model’s cross-species applicability, an additional 70 feline imaging cases were obtained from the hospital archives. These companion animal datasets added anatomical variation and imaging diversity, allowing for a better evaluation of the model’s generalizability. Furthermore, approximately 4000 human limb radiographs were retrieved from public databases including MURA and CheXpert [6,7]. These data provided a foundation for transfer learning, allowing the model to learn high-level fracture detection features from a large and well-annotated dataset. All datasets were anonymized.

### 2.2. Preprocessing and Annotation

Prior to training, images were standardized to 224 × 224 pixels and preprocessed to improve diagnostic features. This included histogram equalization to normalize the contrast, Gaussian filtering to reduce noise, and image cropping or masking to isolate the fetlock joint or other relevant anatomical regions utilizing image processing libraries in Python. To increase data diversity and model robustness, synthetic image transformations like rotation, flipping, brightness variation, and zooming were used.

Histogram equalization was applied using OpenCV’s *cv2.equalizeHist* function on greyscale radiographs to standardize intensity distributions across scanners and exposure settings. This step was applied consistently to all radiographic inputs prior to augmentation. Histogram equalization was used to normalize contrast across radiographs, compensating for differences in exposure or equipment settings. This improved the visibility of subtle features like trabecular changes and early fracture lines. Gaussian filtering was used to reduce imaging noise while maintaining structural edge clarity, thereby improving signal quality and reducing distractions that could mislead the model during training.

In addition to improving visual features, preprocessing aimed to streamline and direct the model’s attention to clinically relevant regions. Cropping and masking techniques were used to isolate the fetlock joint or target anatomical area, removing background information and keeping the model focused on the region of interest. Data augmentation techniques, such as random rotations and horizontal flips, were used to simulate real-world variability in imaging conditions, which is especially useful in veterinary datasets with limited sample sizes.

All fracture regions were manually found and evaluated by experts in veterinary diagnostic imaging (DB and FLD), and the majority of the regions were manually annotated using bounding boxes to define the areas of interest. Annotations were saved alongside metadata indicating the modality (XR, CT, MRI), anatomical region, projection angle, and fracture type. These annotations were critical for both classification and localization training tasks.

### 2.3. Model Architecture and Classification Pipeline

The framework developed in this study has a modular, multi-stage architecture that is specifically tailored to the challenges of veterinary imaging, which involves significant anatomical variability and modality heterogeneity. The first stage of the pipeline focuses on imaging modality classification, which is critical for determining the best downstream processing route. For this task, a ViT integrated with ResNet layers was used, combining ViT’s global attention mechanisms with ResNet’s localized feature extraction strength. For this task, a hybrid Vision Transformer (ViT)–ResNet architecture was employed, combining the global contextual modelling of ViT with the strong local feature extraction of convolutional residual networks.

The ViT component was based on the ViT-Base architecture. Input radiographs were divided into non-overlapping patches of size 32 × 32 pixels, each of which was flattened and linearly projected into a 768-dimensional embedding space. Positional encodings were added to preserve spatial information prior to transformer processing. All experiments used a ViT-Base configuration with patch size 32 × 32 and embedding dimension 768, following the standard formulation proposed by Dosovitskiy et al. [14]. Self-attention was computed using the canonical scaled dot product attention mechanism. Given an input sequence of patch embeddings $X\in R^{N\times d}$, query (Q), key (K), and value (V) matrices were obtained via learned linear projections,

$$Q={XW}_{Q},K={XW}_{K},V={XW}_{V}$$

where $W_{Q},W_{K},W_{V}\in R^{d\times d_{k}}$. Attention was then calculated as

$$Attention(Q,K,V)=softmax(\frac{QK^{T}}{\surd d_{k}})V$$

This standard formulation is included for completeness; the model follows the canonical transformer design and does not introduce modifications to the attention mechanism, consistent with Vaswani et al. [14].

This hybrid model was further improved with adversarial training, which introduces perturbations during learning to increase the model’s robustness to domain shifts and minor input noise, essential when dealing with variable quality veterinary radiographs or scans. To address class imbalance and improve the classification of under-represented modalities (for example, MRI), a custom loss function combining categorical focal loss and standard cross-entropy was employed. Categorical focal loss (Equation (1)) dynamically reduces easy examples and focuses training on difficult-to-classify cases, increasing model sensitivity to rarer classes.

(1) $$L_{focal}=-\sum_{i=1}^{N}\alpha_{i}{\left(1-p_{i}\right)}^{\gamma }y_{i}log(p_{i})$$

where N is the number of classes, $y_{i}\in \{0,1\}$ denotes the ground truth label for class i, $p_{i}$ is the predicted probability for class i, $\alpha_{i}$ is a weighting factor to balance class importance, and γ is the focusing parameter that down-weights well-classified examples to emphasize harder, under-represented cases.

The second module in the architecture was created to handle the view or projection classification of radiographic inputs, which is especially important in equine imaging where dorsopalmar (DP), lateromedial (LM), and oblique views can differ significantly in both appearance and diagnostic interpretation. For this task, a UNI Foundation Model was used. UNI is a lightweight and general-purpose convolutional neural network. This model was trained with active learning, which involved iteratively reintroducing uncertain or misclassified samples into the training set using entropy-based uncertainty sampling (Equation (2)). This approach made better use of a small, annotated dataset and improved generalization across non-standard imaging projections.

(2) $$L_{entropy}=-\sum_{i=1}^{N}p_{i}\left(x\right)log(p_{i}\left(x\right)),$$

where $L_{entropy}$ represents the uncertainty score for the sample x, N is the number of classes, and $p_{i}(x)$ is the predicted probability that sample x belongs to class i. Higher entropy values indicate greater uncertainty, and such samples were prioritized for reintroduction into the training set during active learning.

The fracture detection network is the pipeline’s third and most important module, responsible for localizing fracture regions across species. A Transformer Autoencoder was used, with a ViT-based encoder capturing global context and a decoder reconstructing and highlighting regions corresponding to structural abnormalities. This design enabled the model to learn spatial relationships in the anatomical structure while also focusing on subtle cues that indicated fractures. To improve interpretability and computational efficiency, the ViT encoder was combined with SqueezeNet, a compact architecture known for maintaining performance with far fewer parameters. The detection model was trained with a custom localization-aware loss function that combined binary cross-entropy loss for classification (fracture vs. non-fracture) and intersection over union (IoU) loss for bounding box regression (Equation (3)). The IoU component penalizes poor overlap between predicted and ground truth regions, promoting fracture localization with greater accuracy. For consistency and to minimize projection-based variability, only dorsopalmar (DP) views were used for fracture localization training and evaluation in the current model, as this projection provided the most consistent anatomical reference across the datasets. This combination ensured that the model not only correctly identified fractures but also localized them with high spatial precision, which is required for clinical utility. Overall, this modular design, integrating modality recognition, projection standardization, and interpretable localization, demonstrates the feasibility of building scalable, cross-species AI frameworks for veterinary diagnostics. It highlights how a structured, stepwise architecture can support model adaptability across diverse imaging contexts while maintaining clinical interpretability.

(3) $$L_{comb}=L_{BCE}+{\lambda yL}_{IoU}\;L_{BCE}=-\left[y\log\left(p\right)+\left(1-y\right)\log\left(1-p\right)\right]\;L_{IoU}=1-\frac{\left|B_{p}\;\cap {\;B}_{gt}\right|}{\left|B_{p}\cup {\;B}_{gt}\right|}$$

where y ϵ {0,1} is the ground truth fracture label, p is the predicted probability, $B_{p}$ denotes the predicted bounding box, and $B_{gt}$ is the ground truth bounding box. $L_{BCE}$ captures classification accuracy (fracture vs. non-fracture), while $L_{IoU}$ measures spatial overlap between prediction and ground truth, and λ is a weighting factor to balance classification and localization. In practice $L_{IoU}$ is applied only to fracture-positive cases (y = 1), since non-fracture images have no bounding box annotation. For non-fracture cases, the model is penalized exclusively by the BCE term, ensuring that bounding boxes are not forced when fractures are absent. This conditional design prevents spurious localization while maintaining accurate classification. The transfer learning framework was used to handle the domain shift between human and veterinary imaging. Initially, the fracture detection model was pretrained on the human dataset, which provided abundant, high-volume image data for foundational training. This phase allowed the model to capture common fracture characteristics like cortical discontinuity, trabecular disruption, and joint misalignment. The pretrained model was fine-tuned using the equine datasets. To strike a balance between transferability and adaptability, early transformer layers were frozen, and later layers were unfrozen and retrained with veterinary data. This strategy made domain adaptation more efficient, allowing the model to fine-tune its understanding of species-specific anatomical features without overfitting to smaller veterinary datasets.

To improve clarity and reproducibility, Algorithm 1 summarizes the overall processing flow of the proposed cross-species fracture detection pipeline. The algorithm provides a high-level overview of the sequential steps used during inference and training, including preprocessing, modality classification, projection identification, and fracture localization.


**Algorithm 1: Cross-Species Fracture Detection Pipeline**

*1: Preprocess input image I*

* a. Apply contrast normalization and noise reduction*

* b. Crop or mask region of interest*

* c. Apply data augmentation during training*

*2: Modality classification*

* a. Extract global and local features using ViT–ResNet*

* b. Predict imaging modality (X-Ray, CT, or MRI)*

*3: Projection classification (X-Ray only)*

* a. Apply UNI-based classifier*

* b. Predict projection view (dorsopalmar, lateromedial, oblique)*

*4: Fracture localisation (dorsopalmar view only)*

* a. Encode image using ViT-based encoder*

* b. Predict fracture probability y*

* c. Predict bounding box B using localisation head*

*5: Loss computation (training only)*

* a. Compute binary cross entropy loss for fracture classification*

* b. Compute IoU-based loss for bounding box regression*

*6: Return final prediction (y, B)*


This modular design allows each stage to be independently evaluated or extended, supporting adaptability across species and imaging contexts.

Each model was trained with PyTorch 2.9.1 on NVIDIA A100 GPUs. The AdamW optimizer was used in the training configuration, with an initial learning rate of 1 × 10^−4^ and a cosine annealing scheduler. A batch size of 32 was maintained for 100 training epochs. The combined loss function used binary cross-entropy for classification and an IoU-based regression loss for localization. Within species, the dataset was divided at the case level into 70% for training, 20% for validation, and 10% for testing (test set), ensuring that the test subset remained completely unseen by the model during both training and hyperparameter tuning. All hyperparameters were fixed across experiments to ensure consistency and reproducibility. AdamW was used with a weight decay of $1\times 10^{-4}$. The combined loss function used equal weighting between binary cross-entropy and IoU terms. No exhaustive hyperparameter search was conducted due to dataset size constraints; values were selected based on the prior literature and preliminary stability testing.

For modalities and projection views, class-specific prediction accuracy was calculated as the proportion of correctly classified samples within each true class (i.e., sensitivity). Evaluations on equine training data were also done separately for veterinary images acquired through either hospital-acquired cases or literature-derived cases. Performance was evaluated at the image (patch) level using accuracy (overall classification correctness), sensitivity (true positive rate for fracture detection), specificity (true negative rate), and the mean IoU score (overlap between predicted and annotated fracture regions). To avoid overfitting, an early stop was applied based on validation loss. To account for variability arising from dataset heterogeneity and patch-level predictions, model performance was summarized using median values with interquartile ranges (IQRs). Metrics including accuracy, sensitivity, specificity, and intersection over union (IoU) were computed across test samples and reported as the median [IQR]. This approach provides a robust estimate of central tendency while reducing sensitivity to outliers, which is particularly important in small and imbalanced veterinary datasets.

## 3. Results

The cross-species deep learning framework was evaluated across three core tasks: modality classification, projection view identification, and fracture detection on DP views with spatial localization.

### 3.1. Imaging Modality and Projection Classification

On the test set comprising equine and feline cases, the model achieved an overall modality classification accuracy of 96.7%, correctly distinguishing between radiographs, CT scans, and MRI images despite differences in image resolution and anatomical structure. A detailed confusion matrix on the test dataset (Table 2) reveals strong performance across all modalities. Radiographs (XR) were classified with 93.9% prediction accuracy (170/181), while all MRI images were correctly identified (140/140), and CT scans showed minimal confusion (40/41 correctly predicted). The sensitivity and specificity results further support the model’s clinical reliability, with 100% sensitivity and 97.3% specificity for MRI, 93.9% sensitivity and 99.4% specificity for radiographs, and 97.6% sensitivity and 98.75% specificity for CT, underscoring the robustness of modality recognition even with variable image quality. Misclassifications primarily occurred between MRI and XR, likely due to overlapping greyscale patterns in lower-resolution scans or implant-related artifacts.

The model achieved a training accuracy of 99.6% and a validation accuracy of 97.2% in identifying fetlock vs. non-fetlock equine images and performed robustly across projections with 97.5% test accuracy (Table 3). The model’s high sensitivity (98.3%) and specificity (96.4%) in distinguishing fetlock from non-fetlock images confirms its suitability as a pre-screening tool to streamline downstream projection and fracture classification tasks. The projection-level confusion matrix (Table 4) highlights strong differentiation between DP and other views, while most misclassifications occurred between LM and oblique views. Notably, while all 34 oblique views were identified correctly, the lower sensitivity for LM projections (53.7%) highlights an area for further improvement, likely due to anatomical similarities and variable positioning in LM views.

Together, these classification modules provided a reliable foundation for organizing and preprocessing heterogeneous veterinary imaging data. The high accuracy achieved across different modalities, projection views, and species confirms the robustness of the modular design. This structure ensures consistent data handling and stable performance across imaging conditions, supporting the downstream fracture detection stage and demonstrating readiness for deployment in real-world veterinary diagnostic workflows.

### 3.2. Fracture Detection Performance and Visual Validation of Localization

Given the diverse sources of equine images, we reported both the training and test results, and the model achieved strong performance across all equine datasets (Table 5). To improve statistical robustness, localization performance was reported using median values with interquartile ranges rather than single point estimates. The median intersection over union (IoU) scores were 0.76 for hospital-acquired training images, 0.71 for literature-derived training cases, and 0.84 for the overall test set, reflecting reliable localization despite differences in scan quality and source variability. Sensitivity ranged from a median of 84.0% to 92.3%, indicating the model’s effectiveness in identifying true fracture cases, while specificity ranged from 78.9% to 86.4%, demonstrating a controlled false positive rate across imaging conditions. These ranges highlight that, although performance varied slightly across image sources, central performance values remained consistently high.

**Figure 2:**
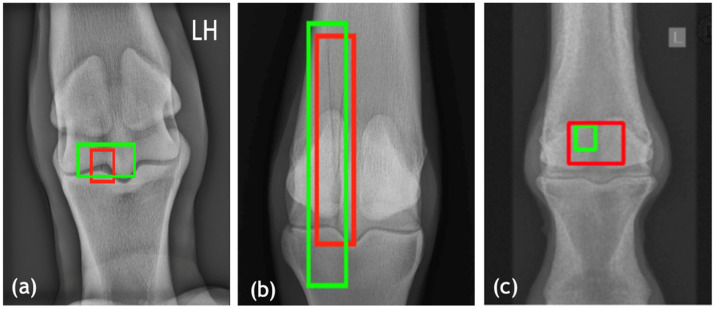
Visual validation of fracture detection in equine datasets for dorsopalmar (DP) projections. Predicted bounding boxes (green) are compared with ground truth annotations (red). (**a**) Hospital-acquired training image with high IoU (0.73) and precise alignment. (**b**) Literature-derived training image with high overlap (IoU = 0.74), reflecting variability in positioning [2]. (**c**) Test set image with high localization accuracy (IoU = 0.81), demonstrating effect of high-quality, standardized imaging protocols.

Exemplars model predictions with ground truth annotations (red boxes) and predicted fracture regions (green boxes) are presented in Figure 2. In hospital-derived training images (example in Figure 2a), the model demonstrated tight bounding box alignment with expert annotations, capturing cortical disruptions and fracture lines with high spatial precision. Literature-derived training images (example in Figure 2b) exhibited slightly lower overlap, likely due to variability in image resolution and positioning. In contrast, the test set images (example in Figure 2c) produced the most consistent and accurate predictions, reflecting the benefits of standardized imaging protocols. However, the model exhibited a degree of over-prediction, with the detected fracture regions extending slightly beyond the annotated ground truth.

**Figure 3:**
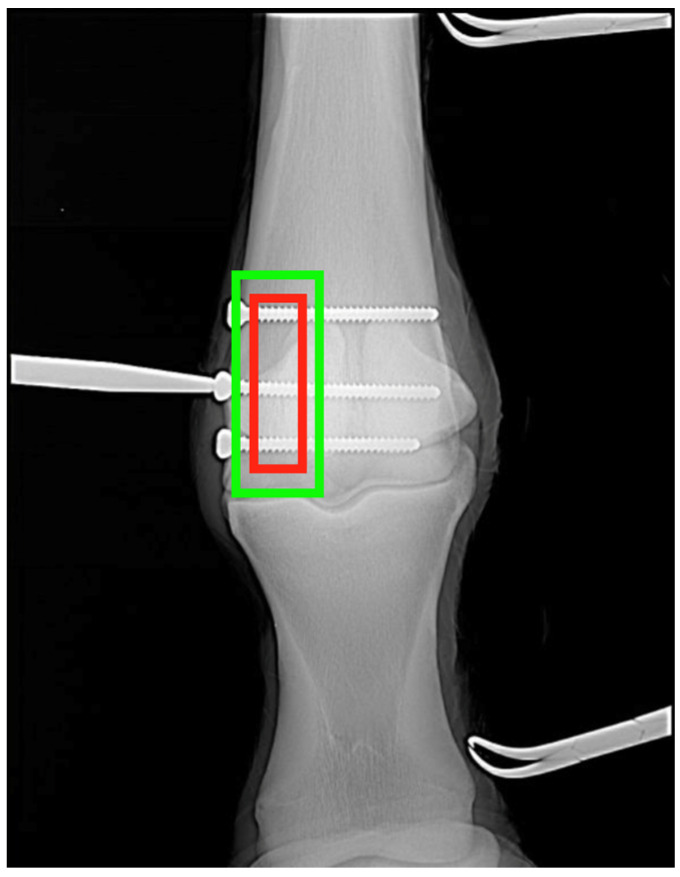
Visual validation of fracture detection in post-operative equine dataset for dorsopalmar (DP) projections. Predicted bounding boxes (green) are compared with ground truth annotations (red).

Prior to fine-tuning using equine images, fracture detection had below 50% accuracy at the image (patch) level on equine datasets, reflecting limited generalization from the initial training phase (pretrained model). After fine-tuning using equine images, the final model achieved high detection accuracy and demonstrated robust generalization across image orientations and scanner types. Performance remained stable when applied to post-operative images containing implants (Figure 3), suggesting resilience to metal artifacts and anatomical distortions. The incorporation of an IoU-based loss function penalizing poor spatial overlap contributed significantly to bounding box precision, which is essential for clinical interpretability. Furthermore, the model’s inference time per image was under 0.5 s on an A100 GPU, making it suitable for real-time triage in clinical settings or batch analysis of hospital archives.

On the human dataset, which the model was originally pretrained on, the final model retained high diagnostic capability after fine-tuning on veterinary images. Following veterinary adaptation, fracture detection accuracy reached 94.1%, with high sensitivity and specificity and good IoU for correctly localized fractures (Table 6). These metrics were calculated per image rather than per case, meaning that each radiograph was assessed independently. These results demonstrate that veterinary fine-tuning did not compromise the model’s original proficiency on human radiographs, where the pretrained model initially achieved 94.1% accuracy and may have further contributed to enhanced generalization through exposure to greater anatomical diversity.

For the feline dataset, the model achieved a good IoU of 0.74 [0.70–0.77], sensitivity and specificity (Table 6). While these results were slightly lower than those observed in the equine test set, performance remained robust across species despite notable differences in bone morphology, joint structure, and image resolution. Importantly, the model was able to localize small-bone fractures, such as those in feline metacarpals, which is typically more challenging due to size and image variability.

**Figure 4:**
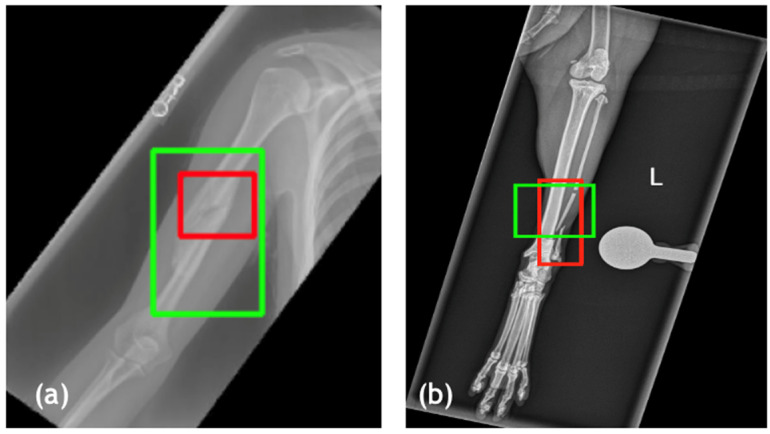
Cross-species validation of fracture detection. Examples from independent test set illustrate model’s generalisability. (**a**) Human radiograph (MURA dataset) with accurate localization (IoU = 0.81). (**b**) Feline radiograph demonstrating reliable localization in small-scale anatomy (ulna of fibula) (IoU = 0.71). Ground truth = red; predicted = green.

To further support the quantitative findings, exemplary visualizations of fracture localization on human and feline radiographs are shown in Figure 4. The predicted bounding boxes (green) closely align with expert annotated ground truth regions (red), demonstrating consistent spatial accuracy across species. In human images, the model reliably detects cortical disruptions and joint misalignments, while in feline images, it successfully identifies subtle fractures in small, morphologically variable bones. The results underscore the model’s ability to detect fractures across species and anatomical contexts, confirming the effectiveness of the transfer learning approach. The diverse pretraining and fine-tuning strategy enabled robust performance on both large-bone (e.g., equine fetlock, human wrist) and small-bone (e.g., feline digit) regions or thin bones (e.g., ulna or fibula).

## 4. Discussion

This study set out to develop and evaluate a modular, deep learning-based pipeline for automated fracture detection in equine fetlock radiographs, addressing a critical diagnostic challenge in veterinary orthopedics. The working hypothesis was that transfer learning from large-scale, annotated human datasets combined with targeted fine-tuning on curated veterinary images could produce a generalizable and high-performing model, even in low-data settings common to veterinary practice. The results of this study not only support this hypothesis but also suggest promising cross-species applicability, particularly in anatomically comparable musculoskeletal regions, suggesting that musculoskeletal imaging tasks may be particularly well suited to shared anatomical representations in artificial intelligence models.

From the perspective of existing research, this work fills a conspicuous gap. While deep learning is now widely applied in human musculoskeletal imaging, particularly for fracture detection in wrist, ankle, and hip radiographs, equivalent tools in veterinary medicine are limited in the literature. Unlike in human medicine, where large-scale open datasets and community challenges such as MURA and CheXpert have driven benchmarking and accelerated progress, veterinary imaging currently lacks any publicly available datasets or competitions. Equine radiographs, and veterinary imaging more broadly, remain fragmented across literature sources and institutional archives, limiting both reproducibility and model development. Prior efforts in veterinary AI have focused on applications such as lameness detection through gait analysis, sound-based respiratory diagnosis, or disease prediction from laboratory tests [9,10,11]. Few, if any, studies have addressed the automated interpretation of diagnostic imaging in horses despite the fact that catastrophic limb fractures, particularly in the fetlock joint, remain one of the most devastating and costly injuries in equine sports medicine. By targeting this clinical area, our study responds directly to an unmet need and provides a replicable blueprint for broader applications in the field.

In the projection classification task, our findings further support the model’s clinical utility. The accurate identification of imaging projections or views is vital in equine radiology, where dorsopalmar, lateromedial, and oblique views provide non-redundant diagnostic information. The UNI-based projection classifier achieved above 97% validation accuracy overall. However, sensitivity varied across views, with notably lower detection rates for lateromedial (LM) projections. This echoes challenges in human imaging, where certain projections obscure key landmarks or create overlapping anatomical features that hinder both manual and automated diagnosis. Addressing this limitation will require expanded datasets and potentially multi-view fusion approaches in future versions of the pipeline.

Our results demonstrate that a transfer learning approach can overcome the limitations of small, fragmented veterinary datasets. Pretraining on over 4000 human radiographs enabled the model to learn generalized fracture features, such as cortical disruption, joint misalignment, and trabecular discontinuity, before being fine-tuned on only equine images. Most of the equine radiographs were literature-derived, supplemented by anonymized hospital archives, reflecting both the scarcity of open veterinary datasets and the need to collate from diverse sources. The model achieved strong localization performance in equine images, and the results are comparable to those reported in leading human studies. For example, Rajpurkar et al. (2017) reported CheXNet achieving over 90% accuracy for pneumonia detection in chest radiographs using a transfer learning strategy [4]. Lindsey et al. (2018) demonstrated that deep learning models could match radiologist-level performance in wrist fracture detection [5], while Kim and MacKinnon (2018) further confirmed the feasibility of transfer learning approaches for musculoskeletal fracture detection [15]. Similarly, Gale et al. (2017) achieved radiologist-level accuracy for hip fracture detection using deep neural networks [16], and Langerhuizen et al. (2019) systematically reviewed AI in fracture detection, highlighting consistent performance gains across multiple anatomical sites [17]. More recently, Costa da Silva et al. (2023) demonstrated promising AI-assisted fracture classification in equine radiographs [18], and Alam et al. (2025) reported robust localization performance in small animal cross-modal imaging tasks [19]. Our findings suggest that the veterinary domain can benefit similarly, provided that curated data, architectural tuning, and domain-specific adaptation are prioritized.

Importantly, the model demonstrated strong performance on companion animals despite never being explicitly trained on feline images during the equine fine-tuning phase. Prior to fine-tuning on equine radiographs, the pretrained model (originally trained on human data) achieved 94.1% accuracy on human radiographs, which was sustained throughout the additional training, but performed poorly on equine images, with below 50% accuracy at the patch level due to domain and anatomical differences. After veterinary fine-tuning, the model maintained its strong diagnostic capability on human images (94.1% accuracy, 95.2% sensitivity, 92.8% specificity, and a median IoU of 0.79 [0.75–0.82]) while achieving comparable performance on equine datasets. This outcome demonstrates that the adaptation process did not compromise human diagnostic performance; instead, exposure to veterinary data improved generalizability by encouraging the model to learn more universal fracture-related features across species. Comparable performance on feline cases (accuracy = 85.9%; IoU = 0.74 [0.70–0.77]; sensitivity = 87.6% [85.0–89.8]; specificity = 84.2% [81.5–86.7]) underscores the effectiveness of the core hypothesis: that fracture-related radiographic features share enough inter-species consistency to enable transfer learning. While formal hypothesis testing was not performed due to limited sample sizes and the clinical focus of this study, the consistent separation of interquartile ranges across datasets supports the robustness of the observed performance improvements. The framework demonstrates improved performance across species, and the learning paradigm is best described as human-to-veterinary transfer learning with fine-tuning, rather than a fully bidirectional training framework.

These results have broad implications. Clinically, this pipeline could be further trained to serve as a decision support tool for equine veterinarians, radiologists, and racing authorities seeking supportive detection tools. Early warning systems based on AI-generated outputs could prompt further imaging, targeted rest, or pre-emptive treatment, thus enhancing animal welfare and reducing loss of use cases. More broadly, this approach provides a methodological foundation for future AI systems in veterinary imaging where data scarcity, imaging variability, and anatomical diversity have historically limited progress.

While the present framework focuses on fracture localization using dorsopalmar (DP) radiographic views, this design choice was intentional. DP projections provide the most consistent anatomical representation of the fetlock joint and offer a stable basis for localization under the constraints of limited and heterogeneous veterinary datasets. Restricting localization to a single, clinically dominant view reduced variability and contributed to the robustness observed across datasets. Future extensions of this work could explicitly model multiple projections (DP, lateromedial, and oblique) as parallel branches, with their outputs combined at a later decision stage to improve sensitivity in cases where fractures are subtle or partially obscured in a single view. Additionally, projection classification and fracture localization could share intermediate feature representations, enabling the joint learning of view-specific fracture patterns rather than relying on fully sequential decisions. From a clinical perspective, incorporating confidence or uncertainty estimates alongside localisation outputs would further enhance usability by highlighting ambiguous cases that may warrant additional imaging or expert review. Finally, while fine-tuning in this study was performed in a single adaptation step from human to veterinary images, future work could explore staged fine-tuning strategies that progressively adapt anatomical representations. This can potentially improve knowledge retention from large human datasets while optimizing performance for animal specific morphology. Although localization performance was evaluated only on DP views in this study, the learned fracture features are expected to remain partially transferable to other projections; however, quantitative generalization to lateromedial or oblique views will require explicit multi-view training and validation, which we identify as an important direction for future work.

Despite these encouraging results, several important limitations should be acknowledged. Despite strong performance across datasets, this study has several important limitations. First, fracture localization was evaluated only on dorsopalmar (DP) radiographic views. While this choice was intentional, DP views provide the most consistent anatomical representation of the fetlock joint, limiting the direct generalization of localization performance to lateromedial or oblique projections. Second, sensitivity was lower for lateromedial views during projection classification, highlighting the challenges posed by anatomical overlap and variable imaging protocols. Third, a substantial proportion of equine radiographs were literature-derived, introducing heterogeneity in image quality, acquisition settings, and annotation standards. In addition, the presence of implants, motion artifacts (which are more frequent in equine imaging, as horses are typically not under general anesthesia), and non-standard projections can all affect localization precision. Finally, although modality classification included CT and MRI, fracture detection was optimized for radiographs only. Further validation on larger, multi-institutional datasets and additional fracture types will be required before clinical deployment.

Future research should prioritize three areas. First, expanding annotated veterinary datasets, particularly including pre-fracture and post-treatment cases, would enable the model to detect subtler pathological changes and move beyond fracture detection towards risk stratification. Second, incorporating multi-view learning, where dorsopalmar, lateromedial, and oblique projections are processed in parallel and fused at the decision stage, may improve sensitivity for fractures that are partially obscured in single views. Third, extending the framework to multi-modality learning using CT and MRI volumes could enhance detection in complex or occult injury cases. Finally, integrating uncertainty estimates and explainable AI tools (e.g., Grad-CAM visualizations) would improve clinical interpretability and support real-world adoption. While receiver operating characteristic (ROC) analysis is commonly used in classification-based medical imaging studies, it was not included as a primary evaluation metric in this work. The proposed framework focuses on fracture localization using bounding box prediction, for which spatial metrics such as IoU, sensitivity, and specificity provide more clinically meaningful assessment. Moreover, evaluation was performed at the image (patch) level rather than at the case level, limiting the interpretability of ROC curves in this setting. Future work, particularly with larger case-level datasets and multi-view aggregation, will enable robust ROC and AUC analyses and facilitate direct comparison with alternative architectures.

## 5. Conclusions

This study presents a modular, cross-species deep learning framework for fracture detection in equine fetlock radiographs, addressing a key gap in veterinary diagnostic imaging. By leveraging transfer learning from large human radiographic datasets and fine-tuning on curated veterinary cases, the proposed pipeline achieved high performance in modality recognition, projection classification, and fracture localization, with strong generalization to feline and human datasets. Importantly, fine-tuning on veterinary images did not degrade human diagnostic performance, supporting the use of human-to-veterinary transfer learning in data-limited settings.

While the current framework is optimized for dorsopalmar radiographs and fracture localization rather than full case-level diagnosis, it provides a robust proof of concept for scalable AI-assisted veterinary imaging. With larger datasets, multi-view learning, and prospective clinical validation, this approach could support decision making in equine sports medicine and contribute to the development of shared benchmarks for veterinary imaging, helping to narrow the technological gap between human and animal healthcare.

## Figures and Tables

**Table 1:** Summary of imaging datasets used in this study.

Species	Cases	Description
Equine images	100	Fetlock radiographs (628 images), computed tomography (CT) (136 images) and magnetic resonance imaging (MRI) (3267 images) collected from the published veterinary literature, equine hospitals and archives, including acute fracture, post-treatment (e.g., with implants), and non-fracture cases. Each case could have multiple images representing different projections.
Feline images	70	Radiographs (70 images) obtained representing a range of limb fractures and non-fracture conditions to assess cross-species adaptability.
Human images	4088	Publicly available human limb radiographs (4088 images) from the MURA and CheXpert databases, annotated for fracture classification and localization, used for pretraining and transfer learning.

**Table 2:** A confusion matrix for modality classification (ViT–ResNet model). The classifier achieved an overall accuracy of 96.7%, with 93.9% sensitivity and 99.4% specificity for radiographs (XR), 100% sensitivity and 97.3% specificity for MRI, and 97.6% sensitivity and 98.5% specificity for CT. Misclassifications were limited and occurred mainly between XR and CT images.

Predicted			
Ground Truth	X-Ray	MRI	CT
**X-Ray (181)**	170	6	5
**MRI (140)**	0	140	0
**CT (41)**	1	0	40

**Table 3:** Confusion matrix for projection classification (UNI-based model). Fetlock vs. non-fetlock classification with 97.2% test accuracy, 98.3% sensitivity, and 96.4% specificity.

Predicted		
Ground Truth	Fetlock	Non-Fetlock
**Fetlock (120)**	118	2
**Non-Fetlock (84)**	3	81

**Table 4:** Confusion matrix for projection classification (UNI-based model) and detailed projection classification (oblique [DMPLO/DLPMO], dorsopalmar [DP], and lateromedial [LM]) showing strong differentiation of oblique and DP views, with reduced sensitivity for LM views due to anatomical overlap.

Predicted			
Ground Truth	Oblique	DP	LM
**Oblique (34)**	34	0	0
**DP (44)**	4	40	0
**LM (41)**	19	0	22

**Table 5:** Fracture detection performance in equine datasets. Results are reported as median values with interquartile ranges (IQRs) to reflect variation across individual images.

Dataset	IoU (Median [IQR])	Sensitivity (Median [IQR])	Specificity (Median [IQR])	Accuracy (Median [IQR])
Training (Hospital-acquired)	0.76 [0.72–0.80]	92.3% [89.5–94.1]	86.4% [82.1–88.7]	89.4% [85.8–91.4]
Training (Literature-derived)	0.71 [0.65–0.75]	84.0% [81.2–86.8]	78.9% [75.3–82.4]	81.5% [78.3–84.6]
Test set	0.84 [0.80–0.87]	88.5% [86.1–91.0]	81.7% [79.0–84.2]	85.1% [82.6–87.6]

**Table 6:** Fracture detection performance across species from 10% test dataset. Results are reported as median values with interquartile ranges (IQRs) to reflect variation across individual images.

Dataset	IoU (Median [IQR])	Sensitivity (Median [IQR])	Specificity (Median [IQR])
Human	0.79 [0.75–0.82]	95.2% [93.0–97.1]	92.8% [90.4–94.9]
Feline	0.74 [0.70–0.77]	87.6% [85.0–89.8]	84.2% [81.5–86.7]

## Data Availability

The original contributions presented in the study are included in the article, further inquiries can be directed to the corresponding author.

## References

[1] Bimson N., Morrice-West A., Wong A., Hitchens P., Rocca M., Whitton R. (2022). Catastrophic Musculoskeletal Injuries in Thoroughbred Racehorses in Uruguay, 2011-2017. J. Equine Vet. Sci..

[2] Santschi E.M. (2008). Articular fetlock injuries in exercising horses. Vet. Clin. N. Am Equine Pract..

[3] Wright I.M., Kidd L., Thorp B. (2022). Fractures in Thoroughbred racing: Epidemiology, prevention, and pre-diagnostic strategies. Equine Vet. J..

[4] Rajpurkar P., Irvin J., Zhu K., Yang B., Mehta H., Duan T., Ding D., Bagul A., Langlotz C., Shpanskaya K. (2017). CheXNet: Radiologist-level pneumonia detection on chest X-rays with deep learning. arXiv.

[5] Lindsey R., Daluiski A., Chopra S., Lachapelle A., Mozer M., Sicular S., Gupta A., Hotchkiss R., Potter H., Westrick E. (2018). Deep neural network improves fracture detection by clinicians. Proc. Natl. Acad. Sci. USA.

[6] Rajpurkar P., Irvin J., Bagul A., Ding D., Duan T., Mehta H., Yang B., Zhu K., Laird D., Ball R.L. (2017). MURA: Large dataset for abnormality detection in musculoskeletal radiographs. arXiv.

[7] Irvin J., Rajpurkar P., Ko M., Yu Y., Ciurea-Ilcus S., Chute C., Marklund H., Haghgoo B., Ball R., Shpanskaya K. (2019). CheXpert: A large chest radiograph dataset with uncertainty labels and expert comparison. Proc. AAAI Conf. Artif. Intell..

[8] Morid M.A., Borjali A., Del Fiol G. (2020). A scoping review of transfer learning in medical image analysis using ImageNet. arXiv.

[9] Nuffel A.V., Zwertvaegher I., Weyenberg S.V., Pastell M., Thorup V.M., Bahr C., Sonck B., Saeys W. (2015). Lameness Detection in Dairy Cows: Part 2. Use of Sensors to Automatically Register Changes in Locomotion or Behavior. Animals.

[10] Oren A., Türkcü J.D., Meller S., Lazebnik T., Wiegel P., Mach R., Volk H.A., Zamansky A. (2023). BrachySound: Machine-learning-based assessment of respiratory sounds in dogs. Sci. Rep..

[11] Bradley R., Tagkopoulos I., Kim M., Kokkinos Y., Panagiotakos T., Kennedy J., De Meyer G., Watson P., Elliott J. (2019). Predicting early risk of chronic kidney disease in cats using routine clinical laboratory tests and machine learning. J. Vet. Intern. Med..

[12] Esteva A., Robicquet A., Ramsundar B., Kuleshov V., DePristo M., Chou K., Cui C., Corrado G., Thrun S., Dean J. (2019). A guide to deep learning in healthcare. Nat. Med..

[13] Wang X., Li X.H., Cho J.W., Russ B.E., Rajamani N., Omelchenko A., Ai L., Korchmaros A., Sawiak S., Benn R.A. (2021). U-net model for brain extraction: Trained on humans for transfer to non-human primates. NeuroImage.

[14] Vaswani A., Shazeer N., Parmar N., Uszkoreit J., Jones L., Gomez A.N., Kaiser Ł., Polosukhin I. (2017). Attention is all you need. Adv. Neural Inf. Process. Syst..

[15] Kim D.H., MacKinnon T. (2018). Artificial intelligence in fracture detection: Transfer learning from deep convolutional neural networks. Clin. Radiol..

[16] Gale W., Oakden-Rayner L., Carneiro G., Bradley A.P., Palmer L.J. (2017). Detecting hip fractures with radiologist-level performance using deep neural networks. arXiv.

[17] Langerhuizen D.W.G., Janssen S.J., Mallee W.H., van den Bekerom M.P., Ring D., Kerkhoffs G.M., Jaarsma R.L., Doornberg J.N. (2019). What are the applications and limitations of artificial intelligence for fracture detection and classification in orthopaedic trauma imaging? A systematic review. Clin. Orthop. Relat. Res..

[18] Costa da Silva R.G., Mishra A.P., Riggs C.M., Doube M. (2023). Classification of racehorse limb radiographs using deep convolutional neural networks. Vet. Rec. Open.

[19] Alam A., Al-Shamayleh A.S., Thalji N., Raza A., Morales Barajas E.A., Bautista Thompson E., de la Torre Diez I., Ashraf I. (2025). Novel transfer learning based bone fracture detection using radiographic images. BMC Med. Imaging.

